# Immunosensor Based on Long-Period Fiber Gratings for Detection of Viruses Causing Gastroenteritis

**DOI:** 10.3390/s20030813

**Published:** 2020-02-03

**Authors:** Marta Janczuk-Richter, Beata Gromadzka, Łukasz Richter, Mirosława Panasiuk, Karolina Zimmer, Predrag Mikulic, Wojtek J. Bock, Sebastian Maćkowski, Mateusz Śmietana, Joanna Niedziółka Jönsson

**Affiliations:** 1 Institute of Physical Chemistry, Polish Academy of Sciences, Kasprzaka 44/52, 01-224 Warsaw, Poland; mjanczuk@ichf.edu.pl (M.J.-R.);; 2 Intercollegiate Faculty of Biotechnology, University of Gdańsk and Medical University of Gdańsk, A. Abrahama 58, 80-307 Gdańsk, Poland; beata.gromadzka@biotech.ug.edu.pl (B.G.); miroslawa.panasiuk@biotech.ug.edu.pl (M.P.); karolina.solarz@biotech.ug.edu.pl (K.Z.); 3 Centre de recherche en photonique, Université du Québec en Outaouais, 101 rue Saint-Jean-Bosco, Gatineau, QC J8X 3X7, Canada; pmikulic@uqo.ca (P.M.); wojtek.bock@uqo.ca (W.J.B.); 4 Baltic Institute of Technology, Al. Zwycięstwa 96/98, 81-451 Gdynia, Poland; s.mackowski@baltech-gdynia.pl; 5 Warsaw University of Technology, Institute of Microelectronics and Optoelectronics, 00-662 Koszykowa 75, Warsaw, Poland; m.smietana@elka.pw.edu.pl

**Keywords:** virus detection, norovirus, virus-like particle, label-free biosensor, optical fiber sensor, long-period fiber gratings

## Abstract

Since the norovirus is the main cause of acute gastroenteritis all over the world, its fast detection is crucial in medical diagnostics. In this work, a rapid, sensitive, and selective optical fiber biosensor for the detection of norovirus virus-like particles (VLPs) is reported. The sensor is based on highly sensitive long-period fiber gratings (LPFGs) coated with antibodies against the main coat protein of the norovirus. Several modification methods were verified to obtain reliable immobilization of protein receptors on the LPFG surface. We were able to detect 1 ng/mL norovirus VLPs in a 40-min assay in a label-free manner. Thanks to the application of an optical fiber as the sensor, there is a possibility to increase the user’s safety by separating the measurement point from the signal processing setup. Moreover, our sensor is small and light, and the proposed assay is straightforward. The designed LPFG-based biosensor could be applied in both fast norovirus detection and in vaccine testing.

## 1. Introduction

Norovirus is the main cause of sporadic and epidemic cases of acute gastroenteritis all over the world. This highly contagious virus with low infectious dose (<10^2^ viral particles) causes illness in all age groups, but incidence rates are the highest among children and older adults [1,2]. In children alone, it results in more than 50,000 annual deaths worldwide [3]. The major barrier in research focused on norovirus detection is the lack of a cell culture system for the multiplication of human norovirus [4]. Although a breakthrough in a method for norovirus cultivation [5] has been recently published, this approach is still rarely used. Additionally, as the norovirus is highly contagious with rapid person-to-person transmission, carrying out research on it is of high risk and requires special facilities and safety equipment. Therefore, norovirus virus-like particles (VLPs) are being used for the development of novel biosensors. VLPs are self-assembled structures composed of multiple proteins that typically form viral capsids. The size and structure are the equivalent or extremely similar to those of the native virus. Due to the lack of genetic material inside the capsid, the VLPs are an attractive and safe alternative to infective viruses in biosensing research. Norovirus VLPs are also widely used as antigens in diagnostic serological assays and as vaccines against norovirus infections [6].

Methods that have traditionally been applied for norovirus detection include reverse transcription-polymerase chain reaction (RT-PCR) and enzyme immunoassays (EIAs), as well as their recently developed varieties [7]. RT-PCR assays are very sensitive, but some strains may not be detected. Moreover, the equipment and reagents are expensive, and the method cannot be used in point-of-care. In turn, EIA assays are fast and highly specific, but their sensitivity remains limited. For those methods, more than 2 h are required to obtain a result. Since a rapid and accurate assay for norovirus detection is not widely accessible, the main diagnostic method is based on the observation of symptoms. Therefore, fast and reliable biosensing methods are strongly needed, and some novel techniques have already been reported. Biosensors for norovirus detection that have been proposed to date are based on electrochemical [8,9,10,11] and optical [12,13] transducers. Recently, the utilization of microfluidic devices was demonstrated for aptamer-based fluorometric and electrochemical detection [14,15]. An interesting approach was proposed by Ashiba et al. [16], where the surface plasmon resonance phenomenon was used to enhance the fluorescence of quantum dots used as labels in the assay. This sandwich assay enabled the detection of 0.01 ng/mL of norovirus VLPs, but it required multiple incubation and washing steps. A very sensitive method (limit of detection = 95 virus copies/mL) involving regeneration of quenched fluorescence of quantum dots/gold nanoparticles upon addition of the virus was also shown in [17]. However, the selectivity of this assay was limited, with high interferences from other virus particles. Another optical biosensor was demonstrated by Chakkarapani et al. [18]. In this fluorescent-free approach, norovirus was detected by immunoreaction with antibody-modified gold nanospots (100 nm) and silver nanoparticles (40 nm), and virus presence was investigated by 3D dual-view light sheet microscopy. The method shows very high sensitivity, but requires unique and advanced optical equipment.

**Figure 1:**
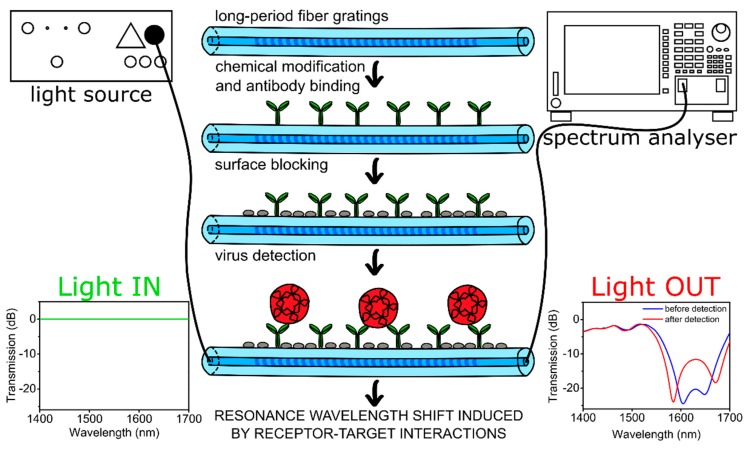
Schematic representation of the experimental setup and fiber surface modification steps leading to norovirus detection. The modifications take place over the whole circular surface of the fiber.

In this work, we report the first application of optical fiber sensors for norovirus detection. The application of optical fiber for signal transmission enables the introduction of a separation in location between the measurement point (i.e., sensor) and the signal processing setup, which may significantly improve user security. As a sensor, we used long-period fiber grating (LPFG), which is a periodic modulation of the refractive index of the fiber core with a period of hundreds of micrometers. This modulation satisfies the phase-matching conditions between the fundamental core mode and forward propagating cladding modes, and therefore several resonances centered at discrete wavelengths appear in the LPFG transmission spectrum. Since the cladding modes’ propagation conditions depend on the external refractive index (RI), it has an impact on the resonance wavelengths. Hence, the LPFG sensor can also be used to monitor the changes in thickness and optical properties of the film formed on the fiber surface. In this way, interactions between target biomolecules and receptors on the grating surface can be detected. Such a detection mechanism, where no fluorescent label is involved, is often referred to as label-free sensing. In addition to high sensitivity, the LPFG does not introduce any additional insertion losses or reflections. LPFG sensors may offer very high RI sensitivity and have already been used as biosensors for the detection of proteins [19,20,21], nucleic acids [22,23], bacteria [24,25,26], and bacteriophages [27]. The main advantages of LPFG biosensors are the real-time and label-free operation, possible miniaturization, and remote sensing capabilities. In our work, the surface of the LPFGs was modified with anti-VP1 antibodies that target the norovirus main capsid protein. The binding events between the VLPs and antibodies were monitored in real time. A scheme of the experimental setup, including fiber surface modifications for norovirus VLPs detection, is shown in Figure 1.

## 2. Materials and Methods

### 2.1. Chemicals and Materials

Bovine serum albumin (BSA), green fluorescent protein (GFP), sodium hydroxide, hydrofluoric acid (HF), ammonium fluoride, acetone, ethanol, 1-ethyl-3-(3-dimethylaminopropyl)carbodiimide hydrochloride (EDC), and phosphate-buffered saline (PBS) tablets were purchased from Sigma Aldrich. The PBS buffer consisted of 0.01 M phosphate buffer, 0.0027 M potassium chloride, and 0.137 M sodium chloride, pH 7.4 at 25 °C. 3-(Triethoxysilyl)propylsuccinic anhydride (TESPSA) and (3-aminopropyl)triethoxysilane (APTES) were purchased from Gelest. Rabbit anti-Norovirus antibodies (ab92976) to Norovirus GII.4 was purchased from Abcam.

### 2.2. LPFG Fabrication and Measurements

The LPFGs with the grating period Λ = 226.8 µm were written in a 5-cm-long section of hydrogen-loaded Corning SMF-28e fiber according to [27]. During the fabrication process, the optical transmission of the fiber was monitored (Yokogawa AQ6370B spectrum analyzer and Leukos SM30 supercontinuum white light laser). After the fabrication, the fiber cladding was chemically etched in order to tune the working point of the LPFG to the dispersion turning point (DTP) of the higher-order cladding modes. The etching was done in 40% HF for 1 min and then in a mixture of 40% HF and ammonium fluoride (1:6 *v*/*v*) [28]. The RI sensitivity of the prepared sensors was measured in glycerol/water solutions with RIs in the range n_D_ = 1.333–1.423 RIU. A Rudolph J57 automatic refractometer was used to verify the RI of the solutions.

### 2.3. Surface Functionalization Methods

To choose the best way for fiber functionalization, we verified a set of surface modification methods. Green fluorescent protein (GFP) was immobilized on the surfaces to check the efficiency of the silanization process. For this purpose, fluorescence confocal microscopy was used. Prior to surface modifications, the optical fiber surface was consecutively cleaned in acetone, ethanol, 0.1 M NaOH, and water (5 min each) and dried under a stream of argon.

#### 2.3.1. Silanization with 3-(Triethoxysilyl)propylsuccinicanhydride (TESPSA)

Silanization was done according to the procedure given in [29] with further modifications. Briefly, the samples were placed in a desiccator over an open container with TESPSA. To evaporate the silane, a pressure of about 1 mbar and additional heating from an externally positioned IR-lamp (Beurer IL 11) was applied. The reaction was continued for 4 h. Then, the sensor surface was cured and annealed to dehydrate the succinic anhydride groups in a furnace at 120 °C for 1.5 h.

#### 2.3.2. Silanization with (3-Aminopropyl)triethoxysilane (APTES)

The samples were placed in a desiccator over two small containers, one containing a 30 μL of the silane precursor (APTES), and the second 10 μL of catalyst—triethylamine—and left at room temperature under an argon atmosphere for 2 h. Next, according to the procedure described in [30], the reagents were removed from the desiccator and the sample was left under an argon atmosphere for curing of the silane layer for 48 h.

#### 2.3.3. Modification with GFP

**Figure 2:**
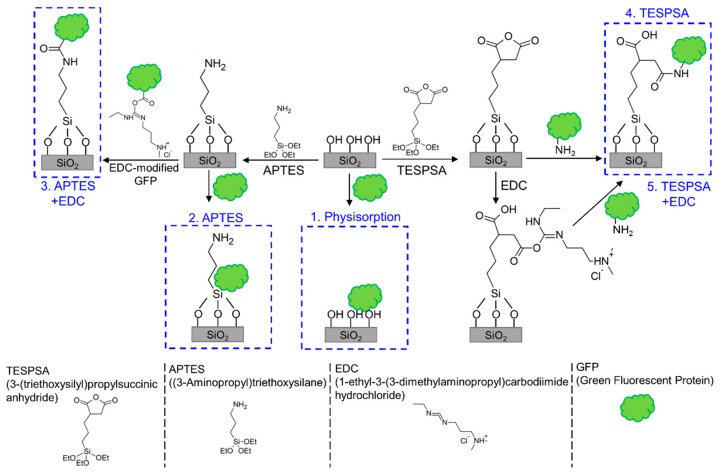
Different methods of surface functionalization for protein receptor immobilization verified in this work.

GFP solution was prepared by diluting the stock solution in phosphate-buffered saline (PBS) to a final concentration 0.01 mg/mL. For one of the tested methods (number 3), carboxylic groups on the surface of GFP proteins were activated with EDC (final concentration 4 mg/mL) for 15 min. Five different methods for GFP immobilization (Figure 2) were verified: (1) physisorption on a clean surface, where samples were incubated in GFP solution for 1 h; (2) physisorption on an APTES-modified surface, where APTES-modified samples were incubated in GFP solution for 1 h; (3) covalent bonding to an APTES-modified surface, where APTES-modified samples were incubated in EDC-activated GFP solution for 1 h; (4) covalent bonding to a TESPSA-modified surface, where TESPSA-modified samples were incubated in GFP solution for 1 h and covalent bonds were formed in a ring-opening reaction; (5) covalent bonding to a TESPSA-modified surface using EDC, where TESPSA-modified samples were first incubated in EDC solution for 15 min to open the succinic anhydride rings and activate carboxylic groups and then incubated in GFP solution for 1 h. All the samples were finally washed extensively with water, dried under a stream of argon, and kept in darkness before the measurements were taken.

### 2.4. Preparation of Biosensor

To prepare the biosensors, we used a covalent bonding to a TESPSA-modified surface. The LPFG sensor was modified with TESPSA according to the procedure described in Section 2.3. During the following steps, the optical transmission of the LPFG in the range of λ = 1500–1700 nm was monitored. The measurements were done in a flow cell (with U-shaped groove, 700 µL) with a polydimethylsiloxane cover, supported by a temperature control system. The temperature was set to 25 °C, and the tension of the optical fiber was kept constant during all the measurements.

LPFG sensor was immersed in anti-VP1 antibody solution (rabbit anti-Norovirus antibodies (ab92976) to Norovirus GII.4, Abcam, 0.01 mg/mL in PBS) for 1 h to covalently bind the antibody to the surface via a succinic anhydride functionality ring-opening reaction that led to the formation of an amide bond. Then, PBS was introduced into the flow system, and measurements were continued for 5 min. This step was repeated three times to remove any unbound antibodies and obtain reference measurements. Next, the BSA solution (0.1% in PBS) was injected into the system for 30 min to block any remaining unspecific sites, and the washing procedure was repeated as described earlier. At the end of sensor preparation, reference measurements in PBS were done.

### 2.5. Norovirus VLP Production and Detection

Norovirus VLP, Rabbit hemorrhagic disease virus (RHDV) VLP, and hemagglutinin protein (HA) VLP (H5N1) were produced in *Sf9* insect cells as described previously [31,32] and purified using ultracentrifugation in sucrose gradient as described in Supplementary Information. The samples were stored at 4 °C prior to use.

The LPFG sensor with anti-VP1 antibody immobilized on the surface was used for the detection of VLPs in solution. For sensitivity analysis, norovirus VLP solutions at concentrations of 1 ng/mL, 10 ng/mL, 100 ng/mL, 1 µg/mL, and 10 µg/mL in PBS were prepared. For selectivity measurements, two negative controls (HA VLP and RHDV VLP) and a positive control (norovirus VLP) were used at a concentration of 10 ng/mL.

During the detection experiments, solutions with increasing concentrations of norovirus VLPs were injected into the stop-flow cell, and the sensor was incubated for 30 min in each solution. After the measurements of each concentration, the sensor was washed and measured in PBS to obtain reference results. The difference between resonance wavelengths before VLPs addition and after VLPs detection was taken as the sensor response. The concentration dependence was checked on three different anti-VP1-modified LPFGs.

Selectivity measurements were done with a similar LPFG sensor modified in the same manner with anti-VP1 antibody and BSA. The sensor was first immersed in non-specific HA VLPs, washed and measured in PBS, then in non-specific RHDV VLPs and in PBS, and finally in specific norovirus VLPs and in PBS. The concentration of all samples was 10 ng/mL, and the incubation time was 30 min.

## 3. Results and Discussion

### 3.1. Sensor Surface Functionalization with GFP

**Figure 3:**
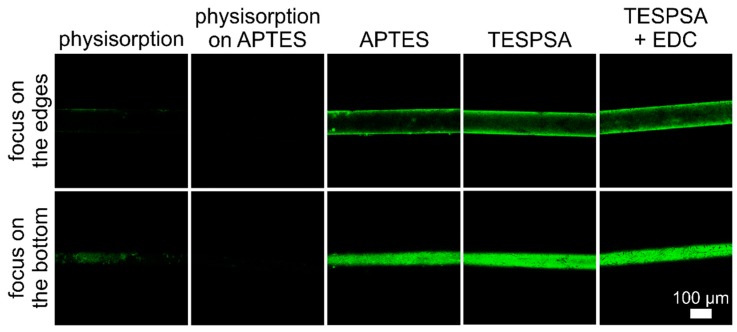
Analysis of different methods of protein immobilization on the optical fiber surface. GFP was used as a model protein.

Immobilization of the receptor (e.g., antibody) on the sensor surface is a crucial step in the biosensor preparation procedure. Therefore, in order to choose an efficient and reliable modification method, we tested immobilization of GFP with different strategies, including physisorption, physisorption on a modified surface, and covalent attachment. For each method, two images were acquired—one focusing on the edges and the other at the bottom of the fiber (Figure 3). As can be observed, physisorption and physisorption on APTES-modified surfaces were not very efficient. In both cases, the quantity and quality of the immobilized protein film (manifested as the intensity and distribution of fluorescence) was low, with a slight advantage of the first approach. The methods with covalent bond formation resulted in good surface coverage. In the immobilization of EDC-activated GFP on APTES, the amide bond formation between proteins and the fiber surface resulted in a high amount of proteins on the fiber surface. However, some protein aggregates were formed due to covalent bonding between EDC-activated carboxylic groups on one protein molecule and amine groups present on the surface of another GFP molecule. Methods with TESPSA and EDC-activated TESPSA resulted in a uniform distribution of GFP on the whole circular surface of the optical fiber, with the highest surface coverage. For the preparation of sensing layers on the optical fiber surface, we chose the method employing TESPSA. As a result, proteins were immobilized on the surface by amide bonds formed in the ring-opening reaction between the succinic anhydride group present in TESPSA and amine groups on the protein surface. The main advantage of this method over the one with EDC-activated TESPSA is the smaller number of steps while maintaining the same performance, which is very advantageous in biosensor development.

### 3.2. Detection of Norovirus VLPs with LPFG Sensor

The RI sensitivity of the LPFG was measured in glycerol/water solutions with RIs in the range 1.333–1.423 RIU and reached almost 2000 nm/RIU for left resonance in RI range 1.333–1.345, as shown in Figure S1 in the Supplementary Information. For biosensing purposes, the LPFG surface was modified with TESPSA according to the selected functionalization procedure. The following experimental steps were done with monitoring of the LPFG transmission spectra in the range λ = 1500–1700 nm at a constant temperature. First, the anti-VP1 antibody was immobilized on the fiber surface, and then 0.1% BSA was used to block the surface and avoid any non-specific interactions. Both steps resulted in a resonance wavelength shift (the left resonance shifted towards shorter wavelengths and the right towards longer wavelengths), indicating successful immobilization of the proteins (Figure S2).

**Figure 4:**
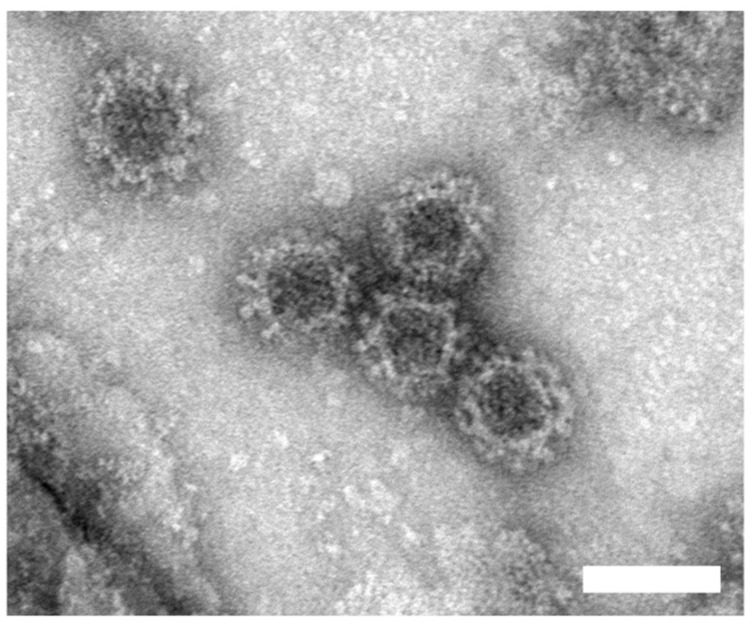
Electron micrographs of purified norovirus VLPs (scale bar: 50 nm).

**Figure 5:**
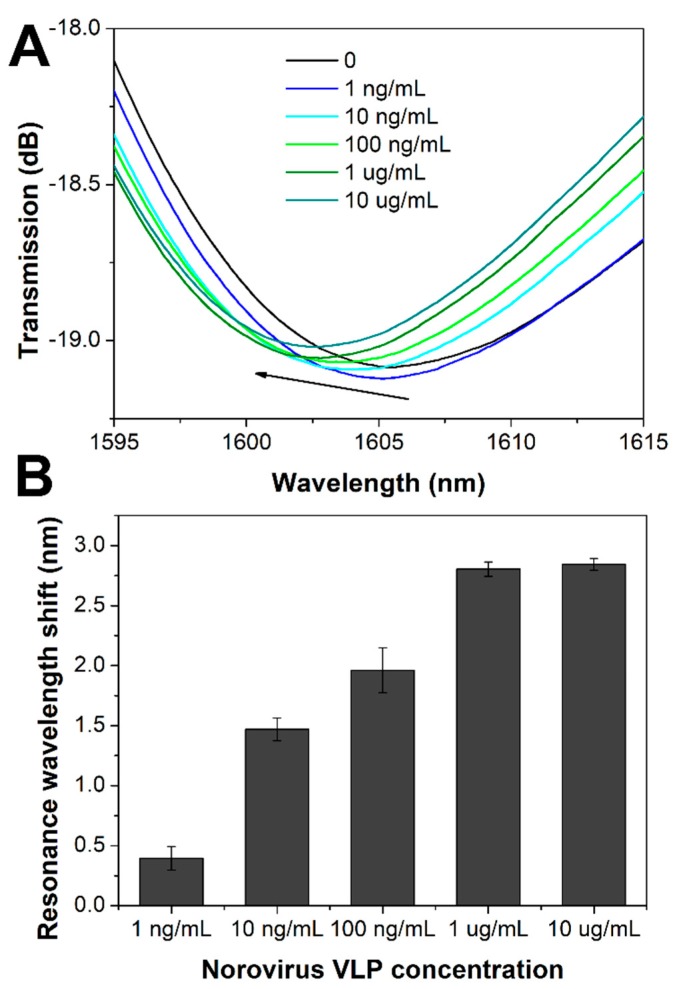
(**A**) Spectra measured in PBS before VLPs addition and after incubation in different concentrations of VLPs. The representative spectra are shown for each concentration. (**B**) Resonance wavelength shift for the left resonance referred to measurement performed in PBS before VLP addition for varied VLPs concentrations.

Norovirus VLPs used for detection were produced in *Sf9* insect cells infected with the recombinant baculovirus. VLPs production, purification and characterization methods, and results are described in the Supplementary Information (Figure S3). The obtained norovirus VLPs were about 40 nm (Figure 4), which corresponds to the size of native norovirus [33]. LPFG sensors modified with antibodies were incubated in solutions with different VLP concentrations ranging from 1 ng/mL to 10 µg/mL. The incubation lasted 30 min, and then the sensor was washed with PBS. As shown in Figure 5A (and Figure S4A), the higher the concentration of VLP, the larger the resonance wavelength shift compared to the measurement done in PBS before detection. The shift for left resonance ranged from 0.4 to 2.8 nm and was caused by the changes in the thickness and optical density of the biolayer formed as an effect of selective norovirus VLPs binding to antibodies. The concentration dependence was repeated on three different LPFG sensors. Each time a significant shift of the resonance wavelength versus noise for 1 ng/mL of added VLP was obtained, and the results are in agreement. Spectral changes observed for VLPs detection were comparable with those obtained for bacteriophage detection with an LPFG sensor [27]. We observed a decrease in sensitivity for higher norovirus VLP concentrations, indicating a possible saturation of receptors with VLPs (Figure 5B).

**Figure 6:**
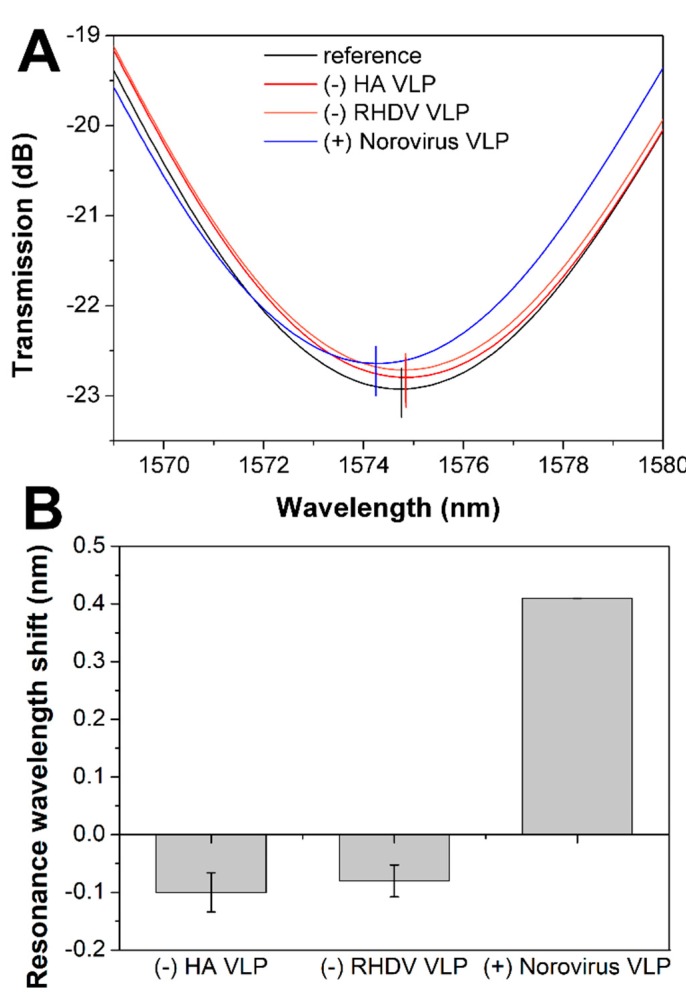
Selectivity analysis of long-period fiber grating (LPFG) biosensor. (**A**) Representative spectra recorded in PBS before specificity test, after incubation in two non-specific VLPs (hemagglutinin protein (HA) VLP and Rabbit hemorrhagic disease virus (RHDV) VLP) and specific norovirus VLPs. (**B**) Resonance wavelength shifts for left resonance for two negative controls (HA VLP and RHDV VLP) and positive control (norovirus VLP).

For selectivity tests, we chose two different VLPs as negative controls—Rabbit hemorrhagic disease virus (RHDV) VLP and HA VLP. The sensor surface was modified with anti-VP1 antibodies as described earlier and subsequently incubated in both negative (10 ng/mL) and positive controls—targeted norovirus VLPs (10 ng/mL) with extensive PBS washing steps between each sample measurement. In the case of negative controls, we observed a slight shift of resonances in the opposite direction compared to the specific binding (Figure 6, Figure S4B). The negative shift might be caused by the detachment of some loosely bound BSA proteins from the surface. Sensor incubation in the positive control caused a resonance shift that confirmed the selective attachment of norovirus VLPs to the anti-VP1-modified surface. Due to slightly lower RI sensitivity of LPFG sensor used for selectivity measurements, the shift for 10 ng/mL norovirus VLP was lower than in the case of previous measurements, but it was still reasonably large.

The LPFG sensor can be reused after the regeneration process, as described in [27].

## 4. Conclusions

The application of an antibody-modified LPFG for norovirus detection enabled us to obtain a rapid, sensitive, and selective biosensor. In this label-free approach, we were able to detect 1 ng/mL norovirus VLPs within 40 min. Such a biosensor may be used in vaccine testing because VLPs are widely studied as possible vaccine candidates against norovirus infections. Moreover, due to the morphological and antigen similarity between VLPs and native norovirus, the LPFG biosensor can be used for fast norovirus detection. The main advantages of the LPFG biosensor for norovirus detection concern simplicity of the measurement and time of the analysis. Future work should focus on the measurements of norovirus samples to confirm the applicability of our sensor design for norovirus infection diagnostics. Additionally, the repeatability and sensitivity of the LPFG sensor may be improved by tuning the dispersion turning point and applying a high-refractive-index coating such as TiO_x_ [34] or TaO_x_ [35].

## Supplementary Materials

The following are available online at https://www.mdpi.com/1424-8220/20/3/813/s1. Materials and Methods—Preparation and characterization of VLPs. Figure S1: Response of the LPFG to external refractive index. Figure S2: Resonance wavelength at subsequent steps of surface biofunctionalization for left resonance. Figure S3: Production and characterization of NoV VLPs in insect cells. Figure S4: Resonance wavelength at subsequent steps of norovirus VLP detection and selectivity measurements.
