# Supplementary material for: Immunosensor Based on Long-Period Fiber Gratings for Detection of Viruses Causing Gastroenteritis
Source: Sensors (Basel). 2020 Feb 3;20(3):813. doi: 10.3390/s20030813 (PMC7038722; doi:10.3390/s20030813)
Supplement: Supplementary file 1 [file sensors-20-00813-s001.pdf]

Supporting Information

Article

Immunosensor Based on Long-Period Fiber Gratings for Detection of Viruses Causing Gastroenteritis

Marta Janczuk-Richter [1], Beata Gromadzka [2], Łukasz Richter [1], Mirosława Panasiuk [2], Karolina Zimmer [2], Predrag Mikulic [3], Wojtek J. Bock [3], Sebastian Maćkowski [4], Mateusz Śmietana [5] and Joanna Niedziółka Jönsson [1,*]

[1] Institute of Physical Chemistry, Polish Academy of Sciences, Kasprzaka 44/52, 01-224 Warsaw, Poland
[2] Intercollegiate Faculty of Biotechnology, University of Gdańsk and Medical University of Gdańsk, A. Abrahama 58, Gdańsk, 80-307, Poland
[3] Centre de recherche en photonique, Université du Québec en Outaouais, 101 rue Saint-Jean-Bosco, Gatineau, QC J8X 3X7, Canada
[4] Baltic Institute of Technology, Al. Zwycięstwa 96/98, 81-451 Gdynia, Poland
[5] Warsaw University of Technology, Institute of Microelectronics and Optoelectronics, Koszykowa 75, Warsaw, Poland
* Correspondence: jniedziolka@ichf.edu.pl; Tel.: +48-22-343-3130

Materials and Methods

Preparation and characterization of VLPs

Production and purification of norovirus (NoV) VLP

The GII.4 NoV 2012 variant (Hu/GII.4/Sydney/NSW0514/2012/AU) *Vp1* DNA coding sequence (1637 bp) was synthesized by Gene Art Gene Synthesis (Thermo Fisher Scientific), and cloned into the baculovirus transfer vector pFastBac1 (Invitrogen, Carlsbad, CA) using EcoRI and NotI restriction sites. For generating a recombinant bacmid DNA, DH10Bac™ competent *Escherichia coli* cells were transformed with the pFastBac1-VP1 transfer vector. Recombinant baculovirus (rBV-VP1) was assembled in *Spodoptera frugiperda* (*Sf9*) insect cells transfected with bacmid DNA. Recombinant baculoviruses containing the *vp1* gene were identified by the immunoperoxidase monolayer assay (IPMA) and by PCR reaction with specific primers based on the pUC/M13 forward and reverse primers.

For the production of NoV VLPs, *Sf9* cells in suspension culture were infected with recombinant baculovirus at an MOI of 3 and harvested 60 h post-infection. Supernatant was collected after centrifugation in a microfuge at 8500 rpm for 10 min.

NoV VLPs were concentrated by ultracentrifugation (Beckman SW-28 rotor) at 82,000 × *g* for 1 h at 4 °C, and pellet was resuspended in NTA buffer (50 mM Tris-HCl, 300 mM NaCl, pH 7.8, 0.1% *v*/*v* Tween 20). One milliliter of concentrated NoV VLPs were loaded onto a 10%–60% discontinuous sucrose gradient and ultracentrifugated at 82,000 × *g* for 1.5 h at 4 °C (SW 41 Ti Beckman rotor) as described before [1,2]. Fractions (1 mL) were collected from the sucrose gradients with thin pipette tips to determine the density and composition of the NoV VLPs samples. Samples were analyzed by SDS/PAGE, Western blotting and ELISA. Purified Nov VLPs were characterized using dynamic light scattering (DLS) and transmission electron microscopy (TEM).

SDS-PAGE

For SDS-PAGE, proteins were dissolved in the denaturing loading buffer and heated for 10 min in 70 °C. Aliquots of 35 μL of each sample were loaded on 10%–20 % precast WedgeWell Gel (Thermo

Fisher Scientific) and run at a constant voltage of 165 V. Polyacrylamide gel was stained with Coomassie brilliant blue solution for 30 min in RT and destained o/n in RT.

Western blot analysis

After semi-dry immunotransfer, PVDF membranes were blocked for 1 h in a 5% semi-skimmed milk solution and incubated overnight with primary antibodies (rabbit anti-Norovirus antibodies (ab92976) to Norovirus GII.4 Abcam) 1:100 in 5% milk solution. The next day, membranes were washed and incubated with secondary goat-anti rabbit-HRP antibodies (1:3000, Jackson) or with secondary goat-anti rabbit-AP antibodies (1:2000, Santa Cruze) solution. Reaction was developed with ECL Plus Chemiluminescent substrate for horseradish peroxidase (Thermo Scientific) or BCIP/NBT stock solution for alkaline phosphatase (Sigma Aldrich).

Dynamic light scattering

Particle sizing was performed using a Malvern Instrument Zeta Sizer NanoS dynamic light scattering instrument (Malvern, Worcestershire, UK). Measurements were taken in water at 25 °C. Sample solutions were passed through 0.45-µm filters and equilibrated at room temperature for 10 min prior to measuring; each measurement duration was 10 s. The results were calculated as the average of six consecutive measurements.

Transmission electron microscopy

The NoV VLPs purified from the supernatant of infected *Sf9* cells were diluted in TM buffer (Tris and $MgCl_2$ buffer). Particles were adsorbed onto carbon-coated grids, stained with 2% uranyl acetate, and examined immediately in a Philips CM 100 electron microscope.

ELISA

A 96-well ELISA plate (Greiner Microlon High-Binding, clear) was coated with 100 µL/well of 14 samples originated from sequential fractions of 0.5 mL after ultracentrifugation. Each sample was ten-times diluted in 1× PBS. The coated plate was incubated o/n at 4 °C. Protein samples were discarded, the plate was washed 4 × 5 min with 200 µL/well of washing buffer (PBS/0.05% Tween 20) and blocked for 1 h – 37 °C/45 min – RT with 250 µL/well of blocking buffer. After 1.5 h of incubation, blocking buffer was discarded and the plate was washed as above. Then, 100 µL/well of primary antibody solution (rabbit anti-Norovirus antibodies (ab92976) to Norovirus GII.4, Abcam) diluted 1:1000 in 3% milk/PBST was added and incubated 1 h, 37 °C. Antibody solution was discarded and the plate was washed as previously. Then, 100 µL/well of secondary antibodies solution (Goat anti-mouse, Jackson ImmunoResearch) diluted 1:1200 in 3% milk/PBST was added and incubated 1 h, RT. Finally, after the last plate washing step (6 × 5 min with 200 µL/well), 100 µL/well of HRP-substrate solution was added (1-Step Turbo TMB- ELISA, Thermo Scientific), incubated in darkness until the desired blue color developed, and stopped by adding 50 µL of 0.5 M sulfuric acid to each well. Absorbance was measured at 450 nm.

Production and purification of negative controls

RHDV VLP

The negative control Rabbit hemorrhagic disease virus (RHDV) VLPs were produced according to [1]. Briefly, the *vp60* gene of polish strain SGM was cloned into pFast Bac1 transfer vector using Not1 and XhoI restriction sites. Recombinant baculovirus was obtained according to manufacturer's instructions (Invitrogen). In order to produce RHDV VLPs, *Sf9* cells were infected with recombinant baculovirus at a MOI of 5 and harvested 60 h post-infection. Supernatant was collected after centrifugation in a microfuge at 8500 rpm for 10 min. VLPs from the supernatant were purified by sucrose gradient (60%–10%) centrifugation at 80,000 × *g* for 1.5 h at 4 °C (SW 41 Ti Beckman rotor).

HA VLP

The extracellular part of hemagglutinin from H5N1 subtype (A/swan/Poland/305-135V08/2006 H5N1-subtype) was produced in *Pichia pastoris* as described previously [3].

Results

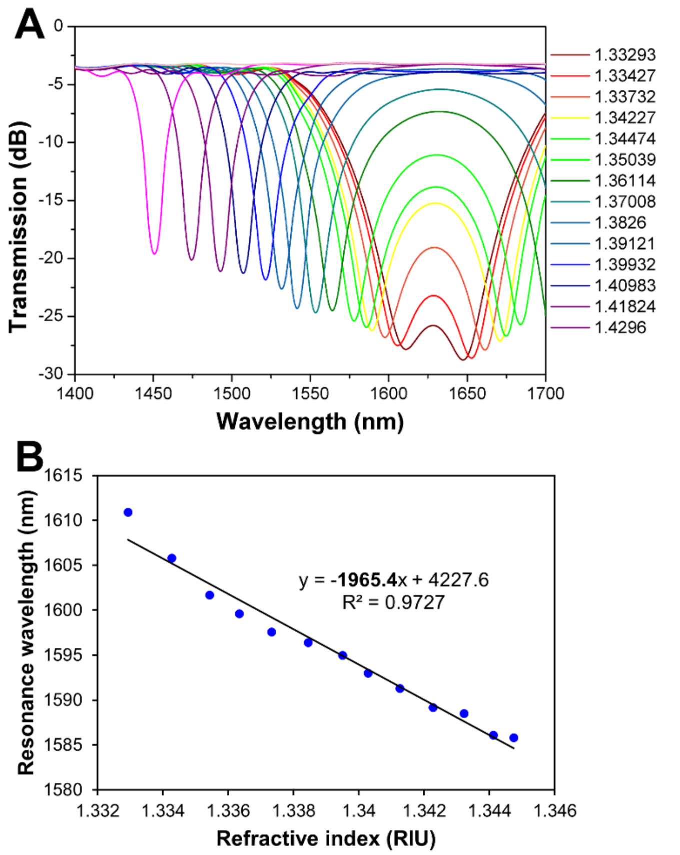

Figure S1. Response of the LPFG to external refractive index. (**A**) Transmission spectra for selected external RIs. (**B**) Resonance wavelength dependence on RI for the left resonance in the RI range 1.333–1.345 RIU.

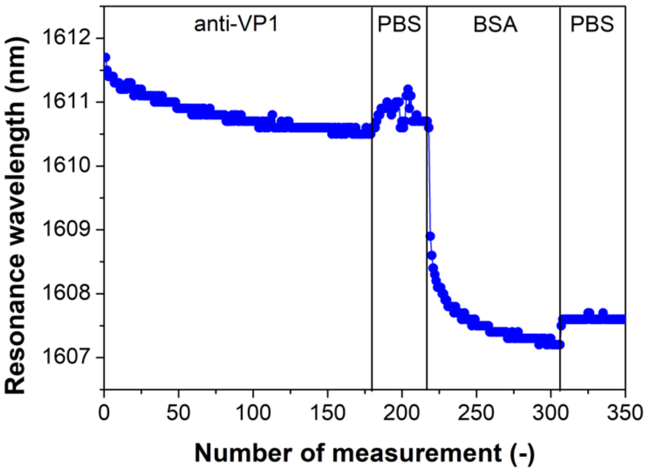

Figure S2. Resonance wavelength at subsequent steps of surface biofunctionalization for left resonance.

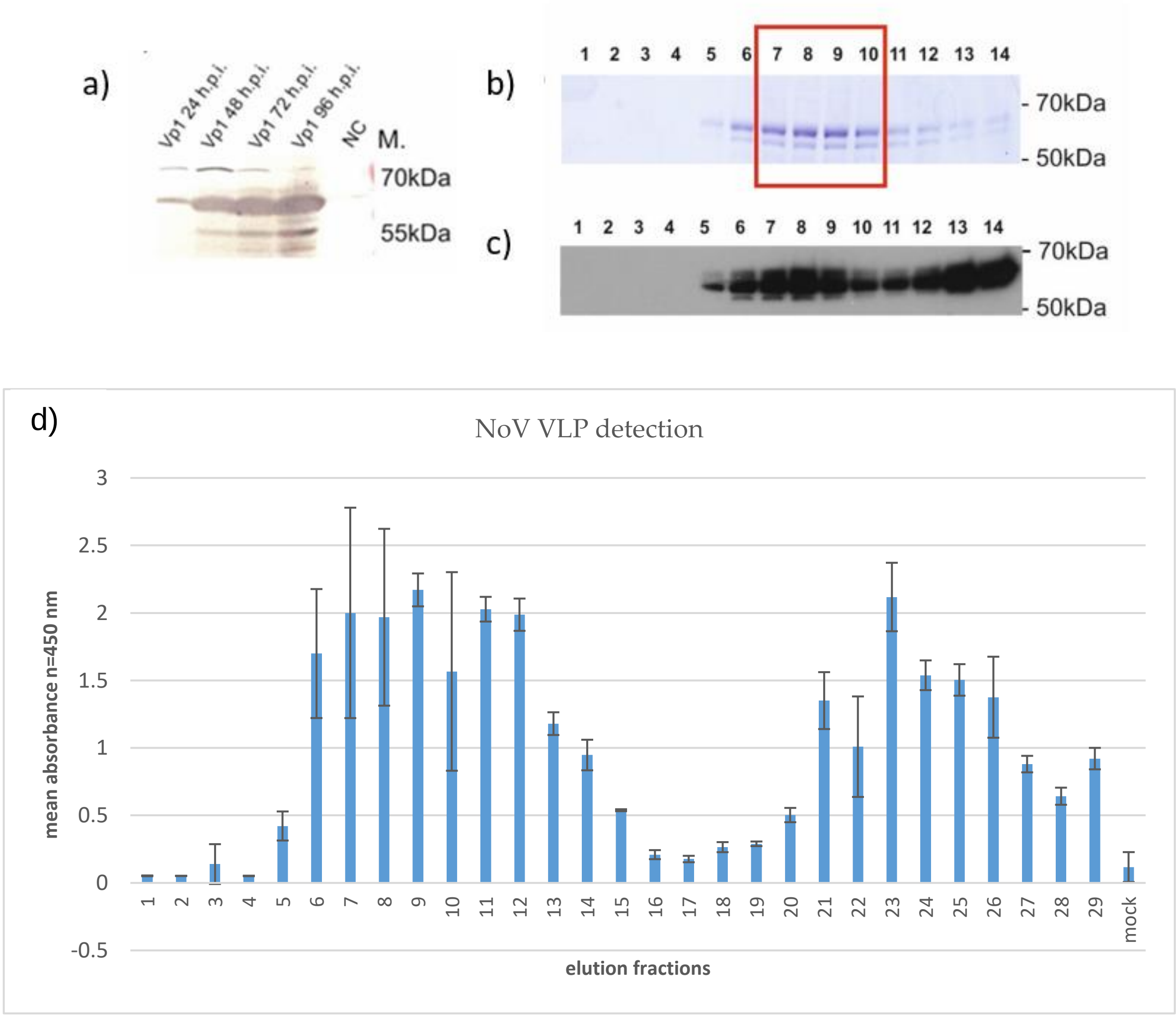

e)

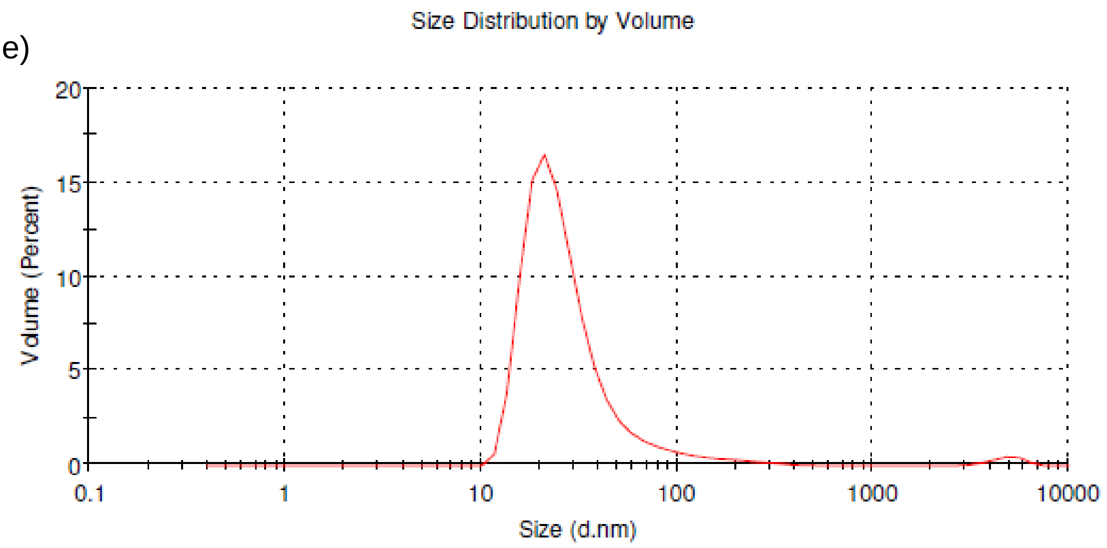

Figure S3. Production and characterization of NoV VLPs in insect cells. (**a**) Time course of VLPs production in insect cells; (**b**,**c**) purification of NoV VLPs using size exclusion chromatography (SDS-PAGE and Western blot); and characterization of NoV VLPs in (**d**) ELISA test and (**e**) DLS.

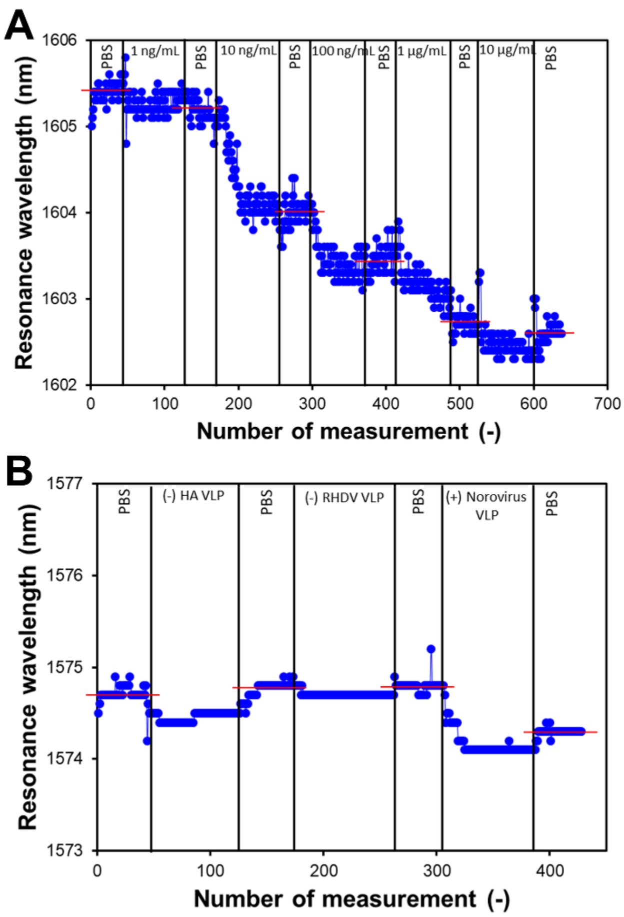

Figure S4. (**A**) Resonance wavelength at subsequent steps of norovirus VLP detection and (**B**) selectivity measurements for left resonance. One measurement took about 23 s.

References

1 B. Gromadzka, B. Szewczyk, G. Konopa, A. Fitzner, A. Kęsy, Recombinant VP60 in the form of virion-like particles as a potential vaccine against rabbit hemorrhagic disease virus *, Acta Biochim. Pol. 53 (2006) 371–376.

2 L. Huhti, V. Blazevic, K. Nurminen, T. Koho, V.P. Hytönen, T. Vesikari, A comparison of methods for purification and concentration of norovirus GII-4 capsid virus-like particles, Arch. Virol. 155 (2010) 1855–1858. doi:10.1007/s00705-010-0768-z.

3 E. Kopera, A. Dvornyk, P. Kosson, K. Florys, V. Sączyńska, J. Dębski, V. Cecuda-Adamczewska, B. Szewczyk, W. Zagórski-Ostoja, K. Grzelak, Expression, purification and characterization of glycosylated influenza H5N1 hemagglutinin produced in Pichia pastoris*, Acta Biochim. Pol., 61 (2014), pp. 597-602.
